# Late presentation of congenital type IV esophageal hiatus hernia in a 9-year-old boy: a case report

**DOI:** 10.1186/s13256-022-03331-9

**Published:** 2022-03-15

**Authors:** Marjan Tariverdi, Zahra Ghaeini Hesarooeyeh, Elham Khalili, Saeedeh Majidi, Maria Rezazadeh

**Affiliations:** 1 Department of Pediatric, Clinical Research Development Center of Children Hospital, Hormozgan University of Medical Sciences, Bandar Abbas, Iran; 2 Student Research Committee, Faculty of Medicine, Hormozgan University of Medical Sciences, Bandar Abbas, Iran; 3 Student Research Committee, Faculty of Medicine, Hormozgan University of Medical Sciences, Bandar Abbas, Iran; 4 Universal Scientific Education and Research Network (USERN), Bandar Abbas, Hormozgan Iran; 5 Assistant professor of Pediatric Surgery, Hormozgan University of Medical Science, Bandar Abbas, Iran; 6 Department of Pediatric, Clinical Research Development Center of Children Hospital, Hormozgan University of Medical Sciences, Bandar Abbas, Iran

**Keywords:** Congenital, Type IV hiatal hernia, Children, Case report

## Abstract

**Background:**

Congenital diaphragmatic hernia affects 1 in every 2000–5000 live births. The mediastinum shifts to the opposite side, the lungs are hypoplastic, and the arterioles are abnormal, resulting in pulmonary hypertension. Respiratory and cardiovascular functions are severely impaired at birth, resulting in significant mortality and morbidity as a result of the associated malformations.

**Case presentation:**

A 9-year-old persian boy was referred with complaint of intermittent abdominal pain in the left lower quadrant and an episode of vomiting. The patient was tachypneic, and the abdomen was nontender on examination. Lung sounds on the left side were considerably decreased, whereas heart sounds on the right side were louder. There was no history of underlying disease in the patient. Initial laboratory blood tests, chest x-ray, spiral computed tomography scan, and chest sonography were requested. Blood tests were normal, and chest x-ray revealed a round-shaped lesion with relatively clear boundaries containing air–fluid level and shift of the heart and mediastinum to the right. A spiral computed tomography scan of the lungs demonstrated the shift of the heart and mediastinum to the right side was due to dilated stomach and colon pressure, and chest sonography revealed that half of the stomach was inside the thorax. Laparotomy surgery was performed. The patient had no complications following surgery.

**Conclusions:**

Herniation of abdominal contents through the diaphragmatic hiatus should be suspected in patients with tachypnea and mediastinal shift to the right side. Rapid diagnosis and early surgical treatment are necessary to avert any potentially life-threatening complications.

## Background

Congenital diaphragmatic hernia (CDH) is defined by a posterolateral muscle defect of the diaphragm, usually (85%) [1, 2] located on the left side, and allows the abdominal viscera to pass into the thoracic cavity.

The Bochdalek hernia accounts for approximately 70% of the cases and occurs in the left posterolateral part of the muscle; the Morgagni hernia accounts for approximately 27% of all cases and involves the anteromedial retrosternal portion of the diaphragm; and the septum transversum-type hernia accounts for approximately 2–3% of the cases. The mediastinum is shifted to the contralateral side in CDH, the lungs are hypoplastic, and the arterioles are anomalous, resulting in pulmonary hypertension. Respiratory and cardiovascular functions are severely compromised at birth, resulting in significant mortality and morbidity due to the associated malformations [3–5].

The estimated congenital diaphragmatic hernia incidence is 1 in 2000–5000 live births [6]. It appears to be slightly more prevalent in males than females and less prevalent in blacks [7, 8].

Congenital diaphragmatic hernia etiology is not understood; however, 2% of cases have been familial, and associated chromosomal abnormalities have been detected in 15% of patients [9].

Hiatal hernias are classified into four types: sliding (type I); paraesophageal (type II); combined (type III), which incorporates elements of types I and II; and giant paraesophageal (type IV) [10].

This case report discusses a late presentation of congenital type IV hiatal hernia.

## Case presentation

**Fig. 1:**
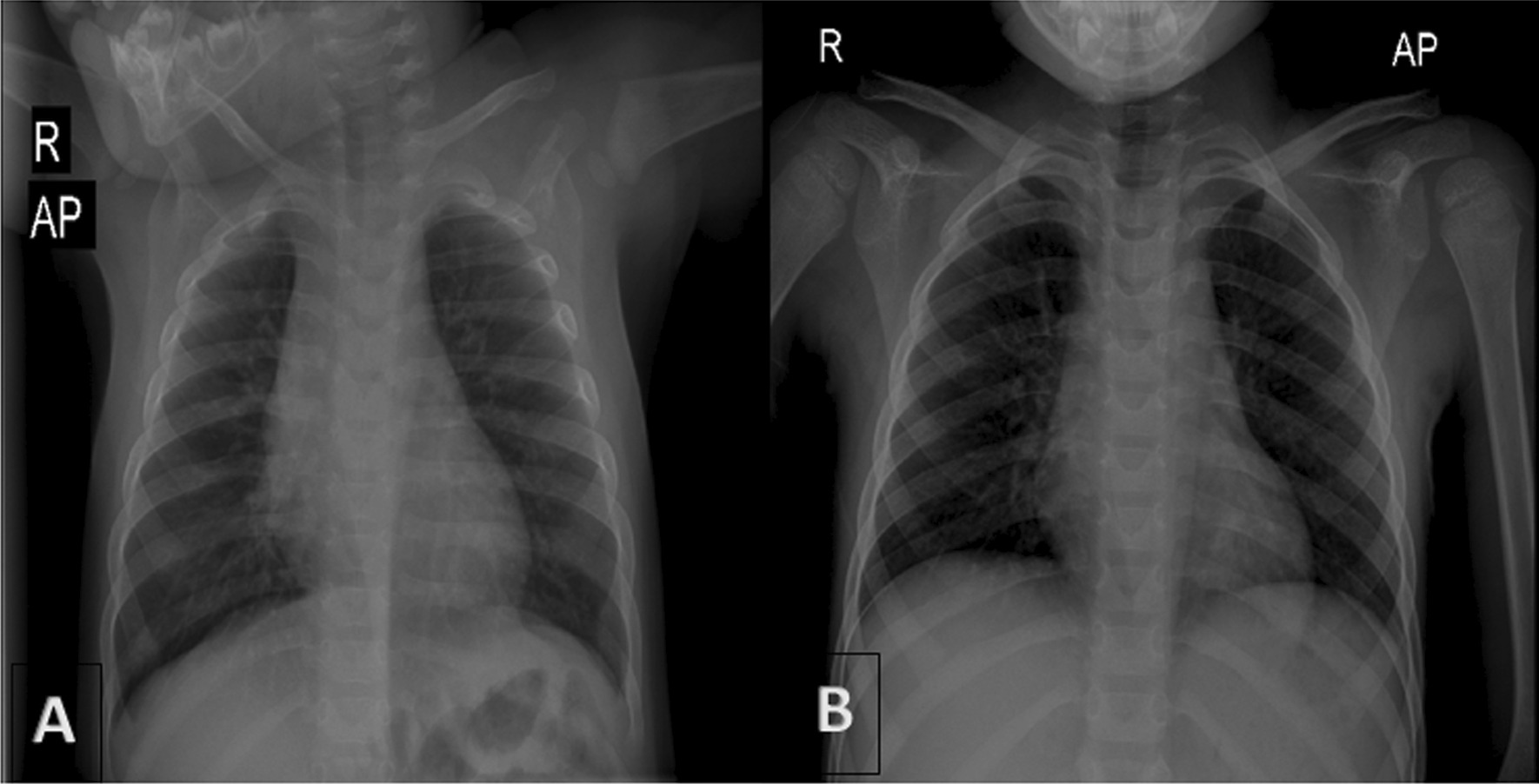
No sign of diaphragmatic herniation on previous CXRs (at age of 11 months, **A**) and (at age of 5 years, **B**)

A 9-year-old persian boy was referred to the emergency department of the children’s hospital complaining of abdominal pain, inconstant in the left lower quadrant area, that started 3 days ago with an episode of nonbilious and nonbloody vomiting. The patient was born by cesarean section without any significant problems, and he had no history of underlying disease. He had been hospitalized twice, once at the age of 11 months because of pneumonia, and once when he was 5 years old owing to fever and cervical lymphadenopathy on the left side; after both admissions, he was discharged home in good general condition. In prior admissions, there was no sign of diaphragmatic herniation on chest x-ray (CXR) (Fig. 1A and B).

**Fig. 2:**
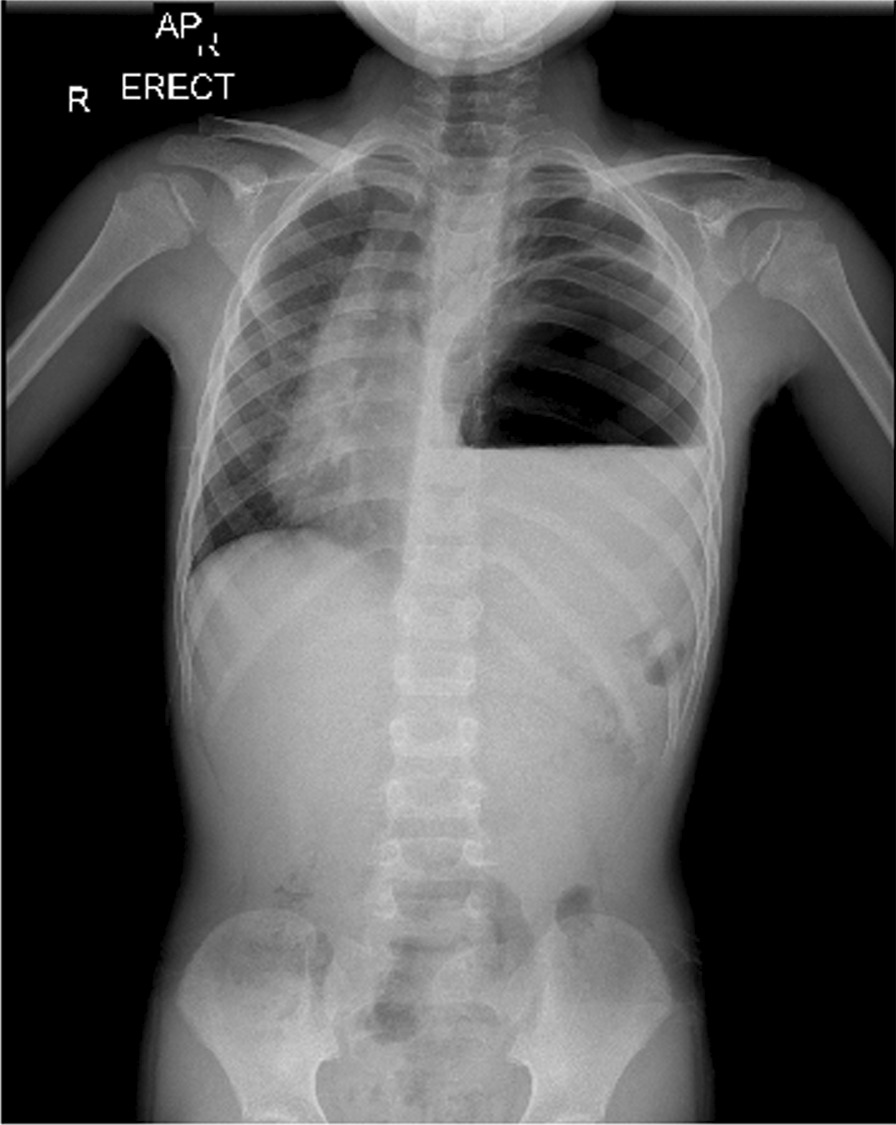
CXR showing a round-shaped lesion with relatively clear boundaries containing air–fluid level, as well as shift of the heart and mediastinum to the right

The patient’s blood pressure (BP) was 135/75 mmHg, pulse rate (PR) was 75 beats per minute, temperature was 36.7 °C, respiratory rate (RR) was 40 breaths per minute, and blood oxygen saturation (SpO_2_) was 99%. On examination, there was a marked decrease in lung sounds on the left side, while auscultation on the right lung was normal. The heart sound was typical and louder on the right side. His abdomen was soft and nontender with no palpable masses, and the rest of the systemic examinations were normal. CXR and initial laboratory blood tests were requested. On CXR, a round-shaped lesion with relatively clear boundaries containing air–fluid level, as well as shift of the heart and mediastinum to the right, was seen (Fig. 2). Hydatid cyst and lung abscess were among our early differential diagnosis based on the CXR. The patient was admitted to the pediatric intensive care unit (PICU) immediately because of mild tachypnea and hypotension. The patient was *nil per os* (NPO) and received oxygen through a nasal cannula; he was continuously monitored by pulse oximetry and cardiac-monitoring device. Intravenous hydration, clindamycin, ceftriaxone, and vancomycin were administered.

Blood tests revealed a white cell count of 8.8 × 10^9^/L (neutrophils 6.6 × 10^9^/L, lymphocytes 1.4 × 10^9^/L); hemoglobin 11.6 g/dL; platelets 312 × 10^9^/L (normal range 150–450 × 10^9^/L); urea 37 mg/dL (normal range 15–45 mg/dL); creatinine 0.46 mg/dL (normal range 0.6–1.1 mg/dL); sodium 144 mmol/L (normal range 135–145 mmol/L); potassium 4.6 mmol/L (normal range 3.5–5.0 mmol/L); prothrombin time 12.5 s (normal range 9.5–12.7 s); activated partial thromboplastin time 36 s (normal range 25.7–38.8 s); International Normalized Ratio 1.1 (normal range 0.9–1.2); C-reactive protein 3 mg/dL (normal range 0–6 mg/dL); erythrocyte sedimentation rate 10 mm/hour. Surgical consultation was also requested for the patient.

Chest sonography illustrated a dilated stomach containing nutrients in the left hemithorax, indicating that half of the stomach was inside the thorax. On abdominopelvic ultrasound, mild free abdominal and pelvic fluid was seen and the rest of findings were unremarkable.

**Fig. 3:**
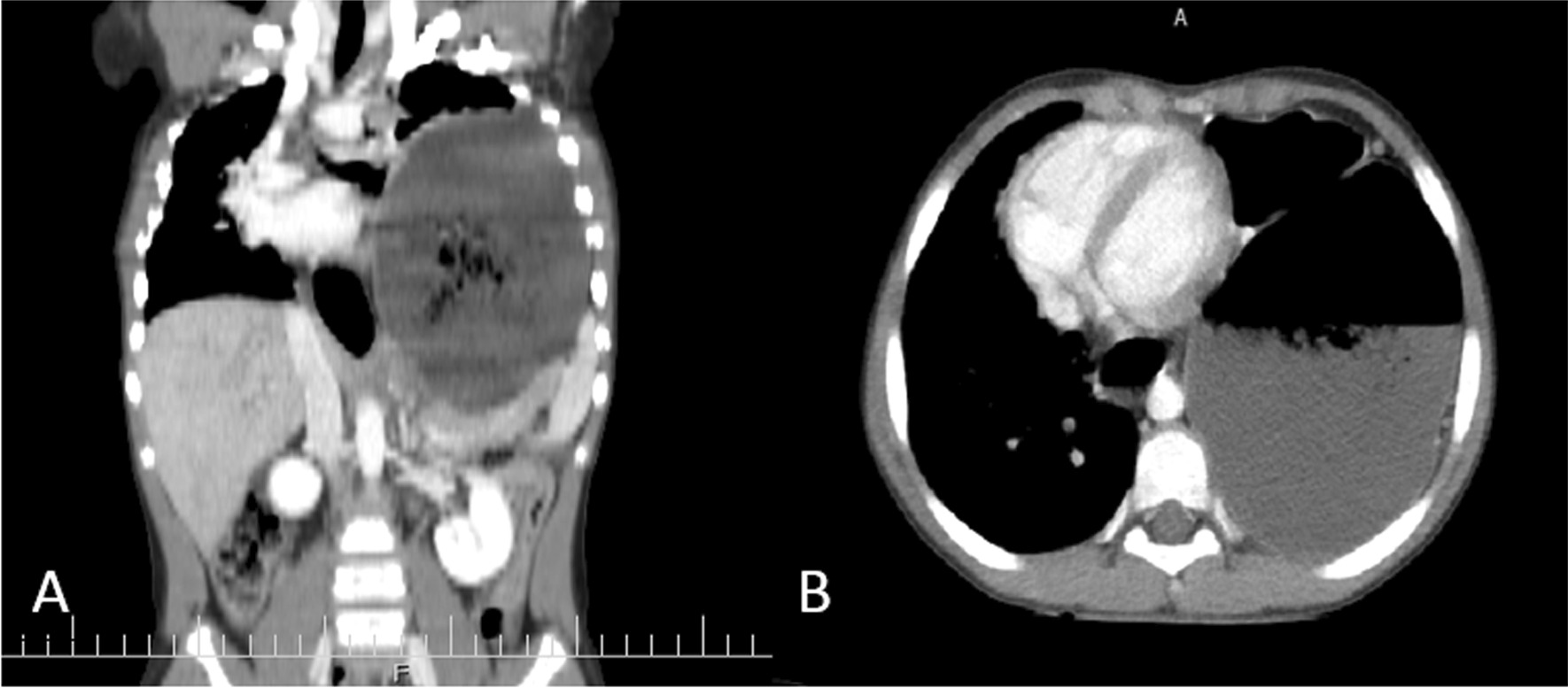
Abdominal CT scan (coronal, **A**; axial, **B**) revealing mild free fluid at the abdominopelvic cavity. Dilated stomach and colon loop are at the left hemithorax

On spiral computed tomography (CT) scan of the lung with intravenous contrast, shifting of the heart and mediastinum to the right side due to pressure effect of a dilated stomach and colon was reported. Colon loop occupying most of the left hemithorax was seen. The esophagus at the distal third was dilated, too, which suggests esophageal hiatal hernia and gastric volvulus (Fig. 3).

**Fig. 4:**
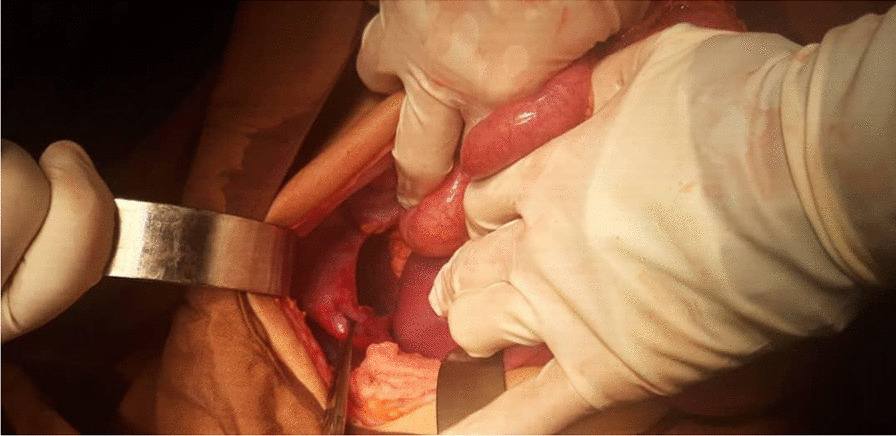
Intraoperation picture. There is a wide defect of 3 cm in the diaphragm in which the stomach, colon, and spleen are inside the thorax

During diagnostic evaluations, the patient had recurrent vomiting. Insertion of a nasogastric (NG) tube was attempted to decompress the stomach, but it did not pass to the stomach and was coiled in the esophagus. He underwent emergent laparotomy because of tachypnea and hypotension. Subcostal incision in the left side used. The diaphragm on the left posterior part had a wide defect of 3 cm, in which the stomach, colon, and spleen were observed inside the thorax, which was reduced to the abdomen meticulously, and the defect was repaired primarily with nonabsorbable sutures. The shape and the location of the defect (which was in the muscular segment of the diaphragm) and edge of the diaphragm resembled a congenital diaphragmatic hernia (Fig. 4). There were no other detectable anomalies such as malrotation.

**Fig. 5:**
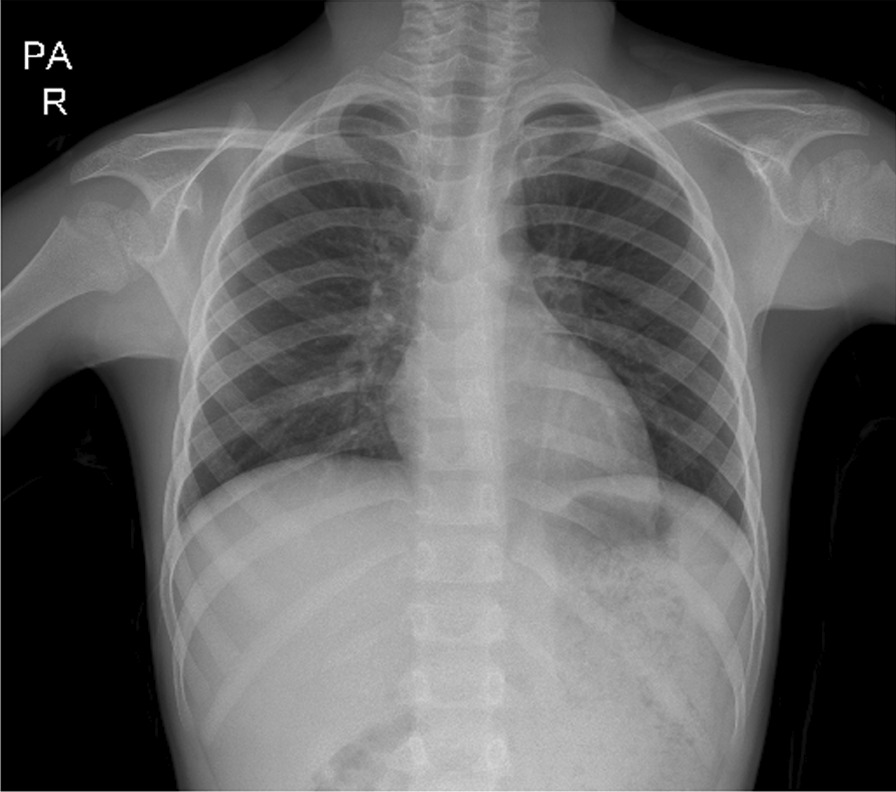
CXR of the patient 1 month after surgery

After surgery, the patient was stable, and hydration and antibiotic therapy were continued. Pantoprazole was administered to prevent stress ulcers. Two days after surgery, the NG tube was removed and liquid diet was started. Incentive spirometry and chest physiotherapy were initiated to rehabilitate pulmonary function. The patient was discharged home uneventfully, and follow-up visits after 3 months showed no complications (Fig. 5).

## Discussion and conclusions

The differential diagnosis of intrathoracic cystic mass should include esophageal duplication cyst, hiatal hernia, pericardial cyst, macrocytic adenomatoid malformation, and neurenteric cyst [11]. As for our patient, initially, two differential diagnoses were made, including lung abscess and hydatid cyst (considering the endemic prevalence). Finally, by performing a CT scan, we reached a definite diagnosis.

The right diaphragmatic crus divides into two branches around the esophagus and forms esophageal hiatus [12]. Hiatal hernia is the protrusion of intraabdominal organs into the thorax [13]. In one study, all the patients with EHH had hiatus widening and exhibited a change of the hiatus shape from sagittal foramen to circular foramen [12]. Another study pointed out that increased body mass index and age is associated with increased prevalence and incidence of EHH [14]. However, our patient was a child with an average body mass index.

Type IV hiatal hernia is extremely rare and accounts for less than 5% of other types of EHH [13]. Type II, III, and IV hiatal hernias account for about 5% of all hiatal hernias, of which type IV hiatal hernias account for 2–5% within that 5% [15]. Type IV hiatal hernia involves herniation of the stomach, omentum, small intestine, colon, spleen, and peritoneum into the thorax [16]. This type of hiatal hernia mainly involves peptic ulcer and acidity, and paraesophageal hiatal hernia is associated with perforation into the stomach [16]. The most common symptoms include chest pain, heartburn, progressive dysphagia, regurgitation, and nausea/vomiting [13]. Our patient presented with only abdominal pain and one episode of vomiting, but later developed hypotension and tachypnea due to mass effect in the mediastinum. During acute phases of hiatal hernia, life-threatening symptoms are observed, including intestinal entanglement, esophageal puncture, bowel obstruction, severe gastroesophageal bleeding, incarceration of the stomach into the thorax, perforation, bleeding, and gastric volvulus [16, 17]. Type IV hiatal hernia is mainly an acquired disorder, arising from repeated episodes of increased intraabdominal pressure with an enlarged diaphragmatic hiatus [17]. Anatomical stressors, including heavy weightlifting, can elevate the intraabdominal pressure, potentially leading to the dislocation of movable abdominal organs through the hiatus into the thorax [17]. There was nothing in our patient history indicative of increased intraabdominal pressure that could predispose to hiatal hernia formation.

There are multiple diagnostic investigations for diagnosing hiatal hernias, but the Society of American Gastrointestinal and Endoscopic Surgeons recommends that only those diagnostic techniques be performed that will affect the patient’s clinical management [18]. The use of some of these techniques can be challenging because of irregularities in the anatomy of the gastroesophageal junction during respiration, movement, and swallowing [19]. Barium swallow, an important diagnostic test for paraesophageal hernia, shows the position and amount of the stomach within the thoracic cavity [20]. Esophagogastroduodenoscopy (EGD) also provides real-time evaluation of the stomach, esophagus, and duodenal mucosa [13]. However, EGD is not able to identify large hiatal hernias [21]. Additionally, CT scans can be used to diagnose hiatal hernia and provide essential details about involved organs and the type of hiatal hernia [13]. In our patient, a type IV hiatal hernia was confirmed on CT scan, on the basis of the displacement of stomach and colon loops through the diaphragmatic hernia.

Owing to dangerous complications, emergent surgery is widely recommended. Indications for surgery include symptomatic patients with paraesophageal hiatal hernia, especially patients with gastric volvulus and obstructive symptoms [19]. Different techniques have been proposed, including different laparoscopic methods and open surgery [17]. Our patient underwent open surgery because of deterioration in vital sign and imminent cardiovascular collapse.

In conclusion, we reported on a late presentation of congenital type IV hiatal hernia in which the stomach, colon, and spleen were found inside the thorax. In patients with abdominal pain and tachypnea, herniation of abdominal contents through the diaphragmatic hiatus should be considered in the differential diagnosis. Plain x-rays are usually diagnostic of the condition, but a CT scan and sonography can confirm it. Rapid diagnosis and primitive surgical treatment are required to avoid any potentially fatal complications, as demonstrated in this case.

## Data Availability

The datasets used during the current study are available from the corresponding author on reasonable request.

## Abbreviations

CDH: Congenital diaphragmatic hernia
CT: Computed tomography
CXR: Chest x-ray
EHH: Esophageal hiatal hernia
NG: Nasogastric
PICU: Pediatric intensive care unit
NPO: *Nil per os*
EGD: Esophagogastroduodenoscopy
